# Combination of artesunate and ruxolitinib suppresses T cell leukemia/lymphoma proliferation via the JAK STAT pathway

**DOI:** 10.1038/s41598-026-39393-8

**Published:** 2026-02-11

**Authors:** Yupei Yuan, Yan Li, Jie Li, Shuyu Wang, Fuyi Luo, Chen Huang, Jie Yang, Yujing Hu, Youchao Jia, Suyun Wang

**Affiliations:** 1 Department of Graduate School, Hebei North University, Zhangjiakou, Hebei, 075000 The People’s Republic of China; 2 Department of Haematology, Hebei General Hospital, Shijiazhuang, Hebei, 050000 The People’s Republic of China; 3 Department of Haematology, The Fourth Hospital of Hebei Medical University, Shijiazhuang, Hebei, 050000 The People’s Republic of China; 4 Department of Nuclear Medicine, Hebei General Hospital, Shijiazhuang, Hebei, 050000 The People’s Republic of China; 5 Department of Oncology, Affiliated Hospital of Hebei University, Baoding, Hebei, 071000 The People’s Republic of China; 6 Department of Haematology, Shenzhen Longhua District Central Hospital, Shenzhen, Guangdong China

**Keywords:** T-lymphoblastic leukemia/Lymphoma, Artesunate, Ruxolitinib, JAK/STAT pathway, Cancer, Oncology

## Abstract

T-cell lymphoblastic leukemia/lymphoma is a highly aggressive malignancy with a 5-year overall survival rate of 35%. Mutations in the JAK/STAT pathway are the second most common mutations in T-cell lymphoblastic leukemia/lymphoma, and hyperactivation of this pathway can promote proliferation of tumor cells. Considering that artesunate and ruxolitinib can inhibit activation of the JAK/STAT pathway, this study investigated the value of these drugs in the treatment of T-lymphoblastic leukemia/lymphoma. Jurkat cells were treated with various concentrations of artesunate and/or ruxolitinib. Cell viability was detected using the CCK-8 assay, and apoptosis was detected after 48 h of treatment using flow cytometry. The effect of each drug alone and in combination was analyzed by polymerase chain reaction and western blotting assays. We also investigated the expression of relevant proteins in the JAK/STAT pathway in lymph nodes and bone marrow samples from 10 patients with T-lymphoblastic leukemia/lymphoma and two healthy controls. Artesunate and ruxolitinib, both alone and in combination, promoted apoptosis and reduced phosphorylation of JAK2 and STAT5 but did not alter mRNA or protein expression of JAK2. The drugs had a stronger inhibitory effect on growth when used in combination than when either was administered alone. Cytotoxicity assays indicated that artesunate had a potent inhibitory effect on cell viability, with IC_50_ values of 12.86 (24 h) and 5.412 µM (48 h), whereas ruxolitinib had weaker activity (with an IC_50_ of 30.55 µM at 24 h and 15.51 µM at 48 h). At 24 h, the total apoptotic rate was 20.75% in the control group, 30.00% in the 20 µM artesunate group, 27.24% in the 30 µM ruxolitinib group, and 43.39% in the combination group. Artesunate and ruxolitinib alone and in combination inhibited aberrant activation of the JAK/STAT pathway in T-lymphoblastic lymphoma/leukemia, supporting the value of this combination in patients with JAK/STAT protein mutations.

**Supplementary Information:**

The online version contains supplementary material available at 10.1038/s41598-026-39393-8.

## Introduction

 The most common subtype of lymphoma/leukemia is acute lymphoblastic leukemia, which accounts for 80% of all cases. Acute T-cell lymphoblastic leukemia/lymphoma (T-ALL), although rarer than that of B-cell origin, is more aggressive^1^. T-ALL is characterized by diffuse infiltration of bone marrow by malignant hematopoietic cells that express markers of immature T-cells^2^. T-ALL accounts for 10%–15% of cases of pediatric acute lymphoblastic leukemia^3^ and 25% of cases in adults^4^. The 5-year overall survival rate for T-ALL has steadily improved in recent decades, exceeding 80% in 2000, mainly because of incremental and strategic changes to multidrug chemotherapy regimens for children and the adoption of pediatric-inspired regimens for young adults^5^. In the 21 st century, the 5-year overall survival rate has reached 85%–90% for children and 40%–50% for adults. However, 30% of patients experience relapse, leading to a poor prognosis^6^ with a 5-year overall survival rate of 35%^7^. Therefore, it is important to explore more potential treatment modalities for T-ALL.

After the NOTCH1 pathway, the Janus kinase/signal transducer and activator of transcription (JAK/STAT) pathway is the second most frequently altered signaling pathway in T-ALL^8^. This pathway plays a key role in the differentiation of helper T-cell subsets and regulation of the immune system and is essential for interferon or cytokine signaling as well as T-cell growth and function^9^. More than 50 cytokines and growth factors are involved in the JAK/STAT pathway, including hormones, interferons, interleukins (ILs), and colony-stimulating factors^10^. Hyperactivation or dysregulation of the JAK/STAT pathway has been linked to immunodeficiency, inflammatory diseases, autoimmunity, and tumorigenesis^9^.

Artesunate is a semisynthetic derivative of the anti-malarial drug artemisinin and has inhibitory effects in several malignancies, particularly leukemia and colon cancer^11^. It can induce apoptosis of T cells in leukemia through the mitochondrial pathway via generation of reactive oxygen species and can also overcome resistance to adriamycin by inducing apoptosis in adriamycin-resistant leukemia cells. Artesunate can promote apoptosis in acute myeloid leukemia by inhibiting activation of the JAK/STAT pathway^12^ and has also been reported to inhibit the proliferation of multiple myeloma through ferroptosis^13^. However, it is unclear whether artesunate has a pro-apoptotic effect in T-ALL in the presence of mutations in the JAK/STAT pathway, as observed in acute myeloid leukemia.

Ruxolitinib, a first-generation JAK inhibitor, mainly inhibits JAK1, JAK2, and JAKV617F and blocks cytokine signaling through the JAK/STAT pathway^9^. Ruxolitinib is a first-line therapeutic option for myelofibrosis and has dramatically improved patient survival^14^. The inhibitory effects of both artesunate and ruxolitinib on the JAK/STAT pathway could permit their use in the treatment of T-ALL with JAK/STAT mutations. Therefore, in this study, we investigated the possibility of combining artesunate and ruxolitinib in the treatment of T-ALL and examined the mechanism of action.

## Methods

### Ethics approval and consent to participate

The cell lines were commercially obtained. All methods were carried out in accordance with relevant guidelines and regulations, including the Declaration of Helsinki. All experimental protocols were approved by the Hebei General Hospital Ethics Committee. Informed consent was obtained from all human subjects and/or their legal guardian(s).

### Immunohistochemistry

Two lymph nodes from patients with lymphadenitis were randomly selected as the negative control group. The experimental group comprised lymph nodes from three patients with T-cell lymphoma and one patient with T-cell lymphoblastic leukemia/lymphoma, alongside bone marrow tissue from six patients with acute T-cell leukemia. All samples were cut into 4-µm-thick sections on positively charged slides (Superfrost Plus, Thermo Fisher Scientific, Waltham, MA, USA). The slides were then baked, dewaxed, and antigenically repaired using thermal induction. Serum closure was performed after blocking endogenous peroxidase. Primary antibodies against JAK2, P-JAK2 (Tyr1007 + Tyr1008), STAT5, and P-STAT5 (all from Abcam, Cambridge, UK) were diluted in phosphate-buffered saline. The sections were then incubated with the antibodies in a wet box at 4 °C overnight. After washing, the sections were incubated with the corresponding secondary antibodies at room temperature for 1 h, and the color was developed with DAB. Nuclei were re-stained with hematoxylin and then dehydrated and sealed. The cells were microscopically examined, and images were captured and analyzed. Hematoxylin-stained nuclei were blue, and positive expression of DAB was brownish yellow.

### Drugs

Artesunate (HY-N0193) and ruxolitinib (HY-50856) were purchased from MedChemExpress (Monmouth Junction, NJ, USA). The initial concentration of artesunate was 100 mM, dissolved in dimethylsulfoxide (MedChemExpress). All drugs used for subsequent treatment of cells were diluted in cell culture medium.

### Cell cultures

Jurkat (acute human T-ALL) cells was purchased from Haixing Biosciences (Jiangsu, China) and maintained at 37 °C in RPMI-1640 medium (Thermo Fisher Scientific) containing 10% fetal bovine serum and 1% penicillin–streptomycin (both from Thermo Fisher Scientific) in an atmosphere of 5% CO_2_ in a cell culture incubator (Thermo Fisher Scientific). The culture medium was changed every 2 days.

### Cell proliferation assay

Jurkat cells treated with various concentrations of artesunate or ruxolitinib alone were divided into single-cell suspensions and inoculated into 96-well plates (20,000 cells/well) with three replicate wells for each time point. After 24 and 48 h of incubation, 10 µL of CCK-8 reagent (Jiancheng Bioengineering, Nanjing, China) was added, after which the cells were incubated for a further 2–4 h. Absorbance was measured at 450 nm using an enzyme-labeling instrument (Bio-Rad, Hercules, CA, USA). Graphs and histograms of Jurkat cell viability were plotted using GraphPad Prism 9.0 software (GraphPad Software Inc., Boston, MA, USA). Jurkat cells were treated with artesunate at approximately 0.5 IC_50_ and the IC_50_ and ruxolitinib at approximately 0.5 IC_50_, IC_50_, and 1.5 IC_50_, either as single agents or in combination. Three replicate wells were used for each concentration. The cells were incubated in a cell culture incubator for 24 h, after which CCK-8 solution was added. Absorbance was measured at 450 nm using a microplate reader. Cell viability was calculated and data were processed using CompuSyn software.

### Apoptosis

Jurkat cells treated with artesunate and/or ruxolitinib were inoculated in 24-well plates at a density of 2 × 10^5^ cells/well and cultured for 24 h. The cells were washed with ice-cold phosphate-buffered saline and suspended in 500 µL of 1× binding buffer. Next, 5 µL of Annexin V-FITC and 5 µL of 7-AAD (both from Elabscience Biotechnology, Houston, TX, USA) were added, followed by incubation at room temperature for 15 min in the dark. The cells were then analyzed using a Cytoflex SRT type flow cytometer (Beckman Coulter, Inc., Brea, CA, USA). Early and late apoptotic cells were identified. The apoptotic rate was calculated using Kaluza Analysis software v2.1 (Beckman Coulter, Inc.)

### Reverse transcription-quantitative polymerase chain reaction

Total RNA was extracted from Jurkat cells using TRIzol reagent (Thermo Fisher Scientific) and treated with Surescript™ First-Strand cDNA Synthesis Kit (FulenGen, Guangzhou, China), followed by processing in a T100™ thermal cycler for reverse transcription into complementary DNA. The complementary DNA was amplified using a reverse transcription-quantitative polymerase chain reaction kit (FulenGen). Complementary DNA was diluted 10-fold as a template, and actin was selected as an internal reference gene for real-time fluorescence polymerase chain reaction (PCR) assays. The PCR protocol consisted of 30 min of amplification at 42 °C, 10 min of denaturation at 95 °C, followed by 40 cycles of denaturation at 95 °C for 15 s and annealing at 60 °C for 1 min. Fluorescence signals were collected, and the relative expression was determined using the 2^−ΔΔCT^ method. The primer sequences are shown in Table 1.

### Western blot assays

Jurkat cells treated with artesunate and/or ruxolitinib were inoculated in six-well plates at 1 × 10^6^ cells/well. Proteins were extracted using RIPA, phenylmethylsulfonyl fluoride, and a phosphoprotease inhibitor (all from Boster Bio, Wuhan, China) after 48 h. A bicinchoninic acid assay kit (Solarbio, Beijing, China) was used for protein quantification. The collected proteins were separated by sodium dodecyl sulfate–polyacrylamide gel electrophoresis at 20 µg per well and transferred to a polyvinylidene difluoride membrane (BiyunTian Biotechnology Co., Ltd., Shanghai, China). After 2 h of blocking using a solution containing skim milk powder, the membranes were incubated with primary antibodies against actin, JAK2, P-JAK2, STAT5, and P-STAT5 (all from Abcam) overnight at 4 °C, followed by incubation with horseradish peroxidase-coupled anti-rabbit secondary antibodies (Boster Bio) for 1 h at room temperature with gentle shaking. Protein bands were visualized using an ultrasensitive luminescent solution (Abbkine, Wuhan, China) and imaged using the ChemiDoc MP system (Bio-Rad). Band intensities were quantified using Image Lab software (Bio-Rad). All primary and secondary antibodies were diluted in antibody dilution buffer (Boster Bio) at the following concentrations: actin, 1:10,000; JAK2, 1:1000; P-JAK2, 1:1000; STAT5, 1:1000; and p-STAT5, 1:1000.

### Statistical analysis

Three independent biological replicates were performed for the cell proliferation and PCR assays, with three technical replicates included in each biological repeat. Three independent biological replicates were used for the western blotting and apoptosis assays. For each biological replicate, the integrated optical density for the target protein in individual treatment samples was normalized to that of the corresponding control samples, after which the ratio of the integrated optical density of the target protein to that of β-actin for the same samples was calculated for correction of the loading error. After confirming the normality of the data distribution, one-way analysis of variance was used for comparisons among multiple groups in the PCR, apoptosis, and western blot assays. The independent-samples *t*-test was used for the cell proliferation inhibition experiment. All statistical analyses were performed using GraphPad Prism 9.0 software (GraphPad Software Inc.). A P-value of < 0.05 was considered statistical significant.

## Results

### Immunohistochemistry

**Fig. 1:**
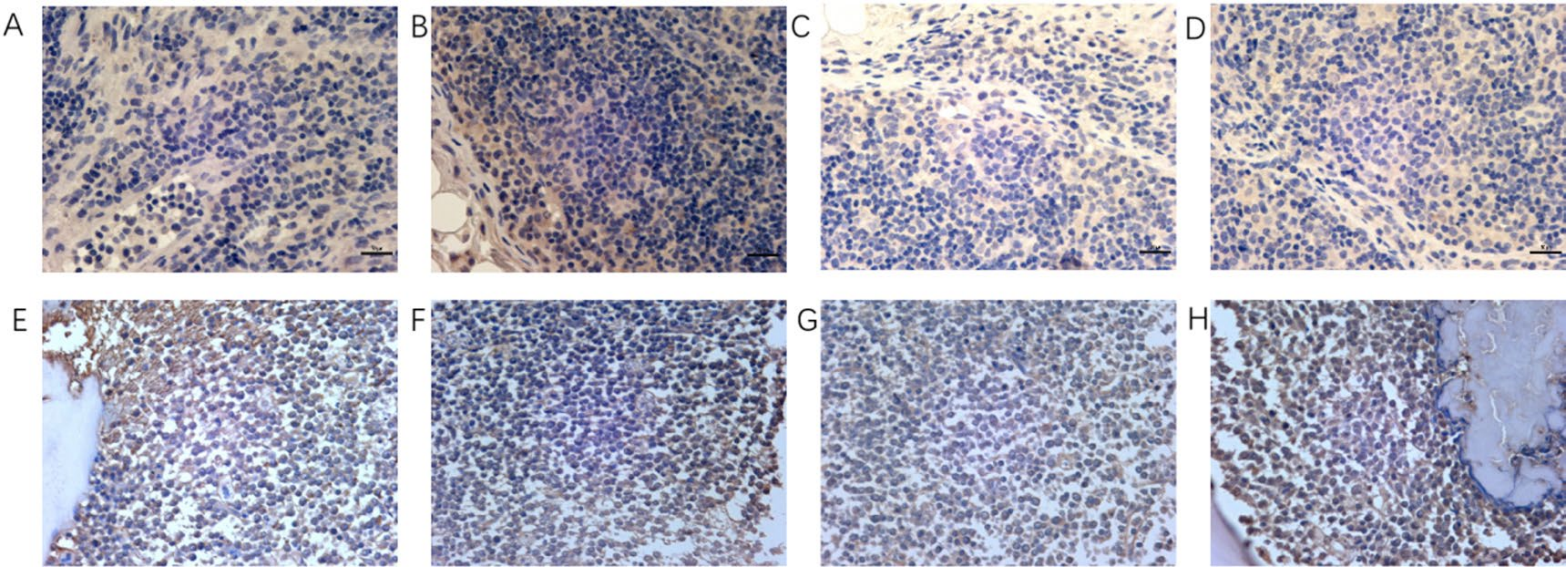
Expression of JAK2, STAT5, P-JAK2, and P-STAT5 proteins in patients with lymphadenitis and T-lymphoma. (**a**–**d**) Protein expression levels in a patient with necrotizing lymphadenitis. (**e**–**h**) Protein expression levels in a patient with acute T-cell lymphoblastic leukemia/lymphoma. Images were captured under a light microscope at 400×magnification.

Lymph node samples from two patients with necrotizing lymphadenitis, three patients with T-cell lymphoma, and two patients with T-cell lymphoblastic leukemia/lymphoma were negative for JAK2, P-JAK2, STAT5, and P-STAT5. Among five patients with acute T-cell leukemia, three bone marrow samples showed positive expression of proteins in the JAK2/STAT5 pathway (Fig. 1).

### Artesunate and ruxolitinib had synergistic effects on growth of Jurkat cells

**Fig. 2:**
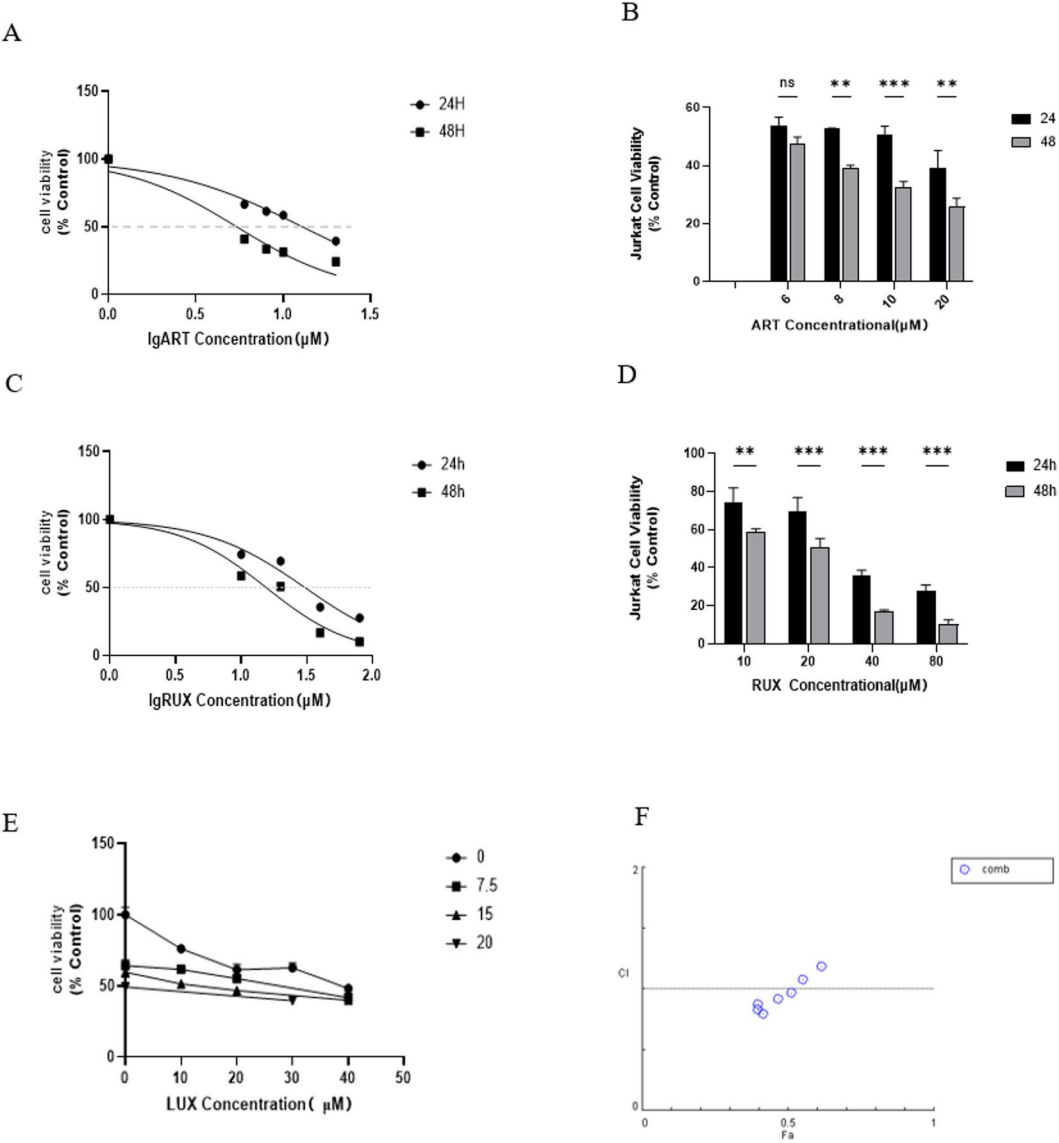
Growth inhibition and concentration–time curves for Jurkat cells treated with various concentrations of artesunate and ruxolitinib (*P* < 0.05). (**a**, **c**) Growth inhibition curves for Jurkat cells treated with 0, 6, 8, 10, or 20 µM artesunate or 10, 20, 40, or 80 µM ruxolitinib for 24–48 h. (**b**, **d**) Analysis of the difference between the two drugs when used for 24–48 h (*P < 0.05, **P *<* 0.01, ***P *<* 0.001, ****P *<* 0.0001). (**e**) Changes in cell viability evaluated following 24 h of treatment with 0, 10, 20, or 40 µM ruxolitinib combined with 7.5 µM artesunate, 0, 10, 20, or 40 µM ruxolitinib combined with 15 µM artesunate, or 20 µM artesunate combined with 30 µM ruxolitinib. (**f**) Combination index graphs for the combination of the two drugs were drawn using CompuSyn software. Combination index > 1, antagonism; Combination index < 1, synergy. ART, artesunate; RUX, ruxolitinib.

The cytotoxic effects of various concentrations of artesunate (0, 6, 8, 10, and 20 µM) and ruxolitinib (10, 20, 40, and 80 µM) on T-ALL cells were evaluated by CCK-8 assay. Artesunate and ruxolitinib decreased Jurkat cell activity versus the control in a concentration-dependent manner, and a stronger inhibitory effect was observed after 48 h than after 24 h (Fig. 2a and d). The IC_50_ of artesunate in Jurkat cells was 12.86 µM at 24 h and 5.412 µM at 48 h, whereas that of ruxolitinib was 30.55 µM at 24 h and 15.51 µM at 48 h (Table 2). Cell viability decreased sequentially after co-treating the cells with 0, 7.5, and 15 µM artesunate and 0, 10, 20, and 40 µM ruxolitinib. The combination index was calculated and plotted using CompuSyn software (Fig. 2e and f). A combination index of < 1 indicated synergistic effects, whereas a combination index of > 1 indicated antagonistic effects. The combination index was > 1 for 7.5 µM artesunate in combination with 10 or 20 µM ruxolitinib, but was < 1 for 7.5 µM artesunate in combination with 10 or 40 µM ruxolitinib. Meanwhile, the combination index was < 1 when 15 µM artesunate was combined with 10, 20, or 40 µM ruxolitinib; similarly, a combination index < 1 was observed for the combination of 20 µM artesunate and 30 µM ruxolitinib (Table 3).

### Artesunate and ruxolitinib can induce apoptosis in Jurkat cells

**Fig. 3:**
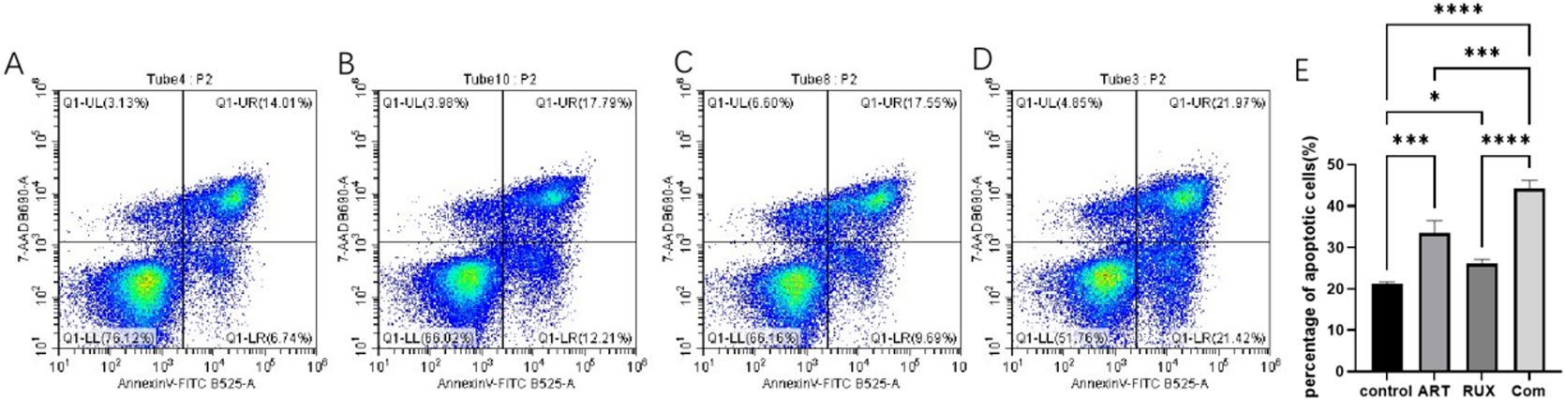
Flow cytometry analysis of apoptosis in Jurkat cells after treatment with artesunate and/or ruxolitinib (*P* < 0.05). (**a**) Blank control group. (**b**) Cells treated with 20 µM artesunate alone for 24 h. (**c**) Cells treated with 30 µM ruxolitinib alone. (**d**) Cells treated with a combination of 20 µM artesunate and 30 µM ruxolitinib for 24 h. (**e**) A statistical analysis chart of the proportion of apoptotic cells after analysis.ART, artesunate; RUX, ruxolitinib; com, combination.

Annexin V-FITC/7-AAD double staining (Fig. 3) revealed that the number of apoptotic cells was significantly increased after 24 h of single-agent artesunate (20 µM) or ruxolitinib (30 µM) compared with the findings in untreated cells. The number of apoptotic cells was further increased by combination treatment with both drugs in comparison with the effect of either drug alone. The proportions of early and late apoptotic cells were 6.74% and 14.01%, respectively, in the control group, 12.21% and 17.79%, respectively, in the 20 µM artesunate group, 9.69% and 17.55%, respectively, in the 30 µM ruxolitinib group, and 21.42% and 21.97%, respectively, in the combination group.

### Artesunate and ruxolitinib can affect RNA expression in the JAK/STAT pathway

**Fig. 4:**
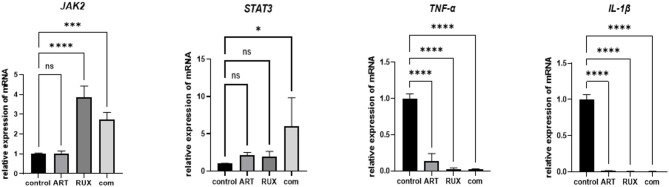
Changes in mRNA expression of differential genes after treatment with various concentrations of artesunate and/or ruxolitinib in Jurkat cells (*P* < 0.05). From left to right, the plots show the relative mRNA expression of *JAK2*, *STAT3*, *TNF-α*, and *IL-1β* (respectively) in Jurkat cells after treatment with the control (untreated), 20 µM artesunate (single agent), 30 µM ruxolitinib (single agent), or the combination (20 µM artesunate + 30 µM ruxolitinib). mRNA levels were quantified by polymerase chain reaction assay and normalized to an internal control. ART, artesunate; RUX, ruxolitinib; com, combination.

RNA was extracted after treating Jurkat cells with 20 µM artesunate and 30 µM ruxolitinib for 24 h to detect changes in *JAK2*, *STAT3*, *IL-1β*, *TNF-α*, and *Actin* RNA expression at the RNA level. A significant difference in *IL-β* and *TNF-α* expression was observed between the two single-drug groups and the two-drug group. *JAK2* mRNA expression did not differ between the artesunate and control groups, but its expression was increased by ruxolitinib and the two-drug combination. *STAT3* expression tended to be elevated when ruxolitinib was used alone in comparison with the effects of the two-drug combination (Fig. 4).

### Artesunate and ruxolitinib can induce death of Jurkat cells by inhibiting phosphorylation of proteins involved in the JAK/STAT pathway

**Fig. 5:**
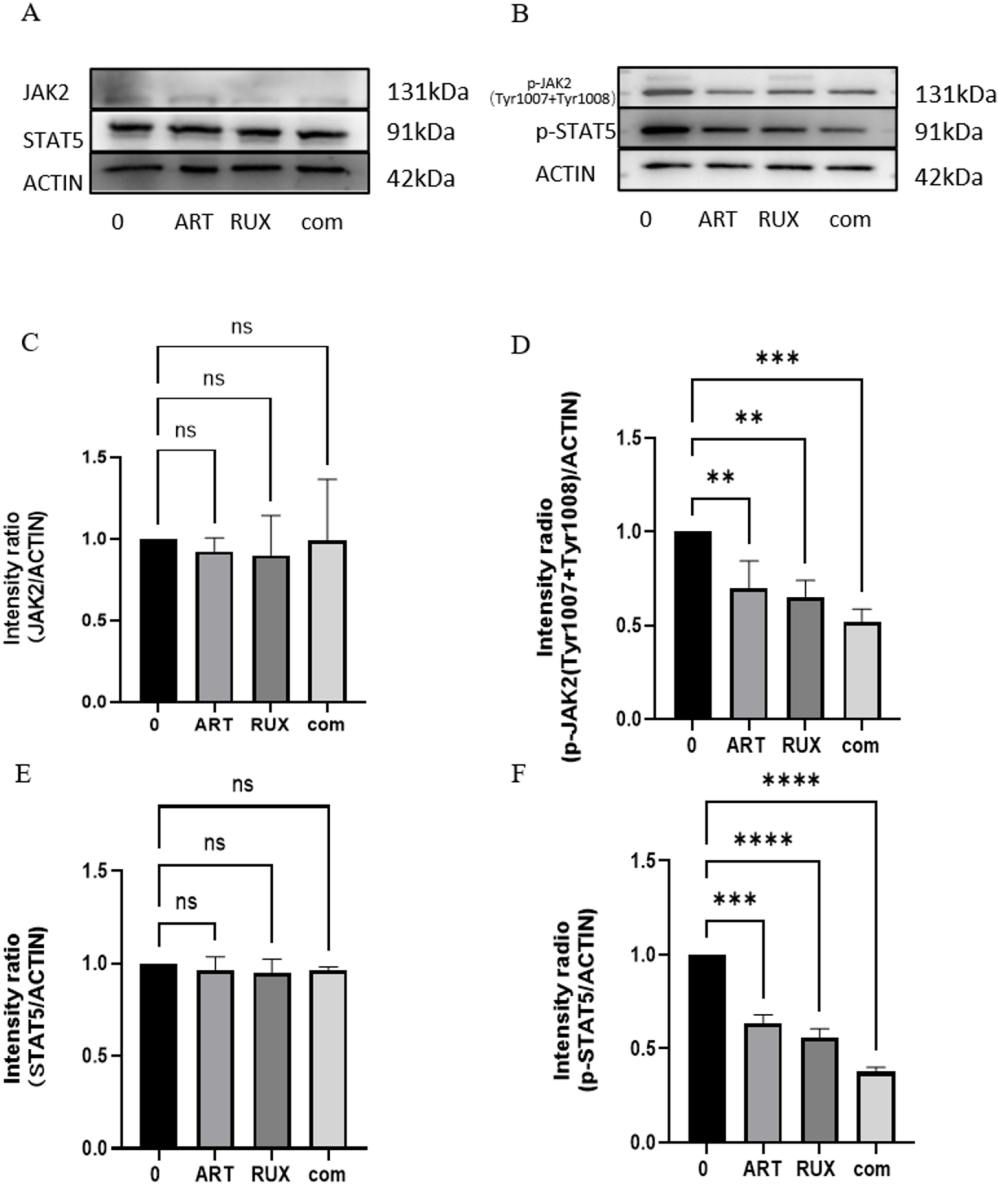
Protein expression of JAK2, STAT5, P-JAK2 (Tyr1007 + Tyr1008), and P-STAT5 after 24 h of treatment with artesunate and/or ruxolitinib in Jurkat cells (P *<* 0.05). (**a**, **b**) Expression of the target proteins JAK2, STAT5, P-JAK2 (Tyr1007 + Tyr1008), and P-STAT5. The internal reference protein β-actin was detected using western blotting after treating Jurkat cells with 20 µM artesunate, 30 µM ruxolitinib, or the combination of 20 µM artesunate and 30 µM ruxolitinib for 24 h. (**c**–**f**) JAK2, STAT5, P-JAK2 (Tyr1007 + Tyr1008), and P-STAT5 expression levels were analyzed as gray values after drug treatment (NS, *P* > 0.05, *P < 0.05, **P *<* 0.01, ***P *<* 0.001).ART, artesunate; RUX, ruxolitinib; com, combination.

Proteins were extracted after treating Jurkat cells with 20 µM artesunate and 30 µM ruxolitinib for 24 h to detect changes in expression of JAK2, P-JAK2, STAT5, and P-STAT5 proteins. At the protein level, JAK2 and STAT5 expression remained unchanged after all treatments, but their phosphorylation levels were reduced by both monotherapy and combination treatment (Fig. 5). These findings indicated that both drugs change the activation state of JAK2 and STAT5 without influencing their expression.

## Discussion

The finding that the JAK/STAT pathway is activated in approximately 25% of cases of T-ALL^2^ is inconsistent with the results of immunohistochemistry. This inconsistency is related to the source of samples and small sample sizes. In one study, JAK/STAT pathway mutations were found in 11 of 28 patients with angioimmunoblastic T-cell lymphoma and in 42% of those with peripheral T-cell lymphoma^15^. The mutation rate of proteins in the JAK/STAT pathway is low, and it was difficult to detect a positive result in the present study, given that the experimental group included only three patients. This sample size was insufficient to observe differences in the positive and negative counts; however, the positive shift in T-ALL provides justification for the use of JAK2 inhibitors. Considering that progression-free survival is poorer in patients with mutations in the JAK/STAT pathway than in those without these mutations^15^, it is clinically important to explore therapeutic regimens for T-ALL with mutations in this pathway. The potential of JAK/STAT pathway inhibition as a treatment strategy for T-ALL can be explored at an early stage by assessing the effects of drugs on Jurkat cells, which were derived from a patient with T-ALL.

The combined application of 15 µM artesunate with 10, 20, 40 µM ruxolitinib and 20 µM artesunate with 30 µM ruxolitinib has a synergistic effect but has an antagonistic effect at low concentrations. At low doses, antagonism arises from drug target competition, insufficient attainment of effective activity thresholds, and compensatory cellular responses; at high doses, synergism is achieved via dual blockade and saturation of the target pathway. A core consideration for clinical translation is whether the concentrations required for synergism can be safely achieved in patients.

The JAK family has four members, namely, JAK1, JAK2, JAK3, and TYK2. JAK3 is expressed in bone marrow, the lymphoid system, and endothelial and smooth muscle cells^16^, while the other members are expressed in almost all tissues^17^. JAK2 mutations are associated with many hematologic malignancies^18^. The STAT family consists of seven members: STAT1, STAT2, STAT3, STAT4, STAT5a, STAT5b, and STAT6. Aberrant STAT5 signaling has been implicated in the pathogenesis of hematologic and solid organ malignancies^19^. STAT5 mutations have been found to enhance colony formation in Jurkat cells^20^.

In the present study, exposure to ruxolitinib increased the RNA expression of *JAK2* in Jurkat cells, whereas only P-JAK2 expression was reduced at the protein level, similar to the findings of Koppikar et al.^21^. Therefore, the core effect of ruxolitinib on Jurkat cells is control of downstream signaling by decreasing phosphorylation of JAK2. The divergence in mRNA and protein expression might be related to reduced phosphorylation of the downstream protein STAT after ruxolitinib-induced inhibition of JAK2 activity, resulting in increased *JAK2* transcription through negative feedback. Ruxolitinib also significantly inhibited transcription of the upstream signals *IL-1β* and *TNF-α*, with no significant effect on *STAT3* transcription noted. Expression of P-STAT5 was reduced by exposure to ruxolitinib; however, STAT5 expression was unchanged, indicating that ruxolitinib altered the activation of STAT5. Combined with the change in the expression of the upstream protein JAK2, ruxolitinib inhibited the growth of Jurkat cells and induced apoptosis by inhibiting abnormal activation of the JAK2/STAT5 signaling pathway. In another study, refractory T-ALL without concomitant JAK1, JAK3, and STAT5B gene mutations was converted to microscopic residual disease in bone marrow after treatment with ruxolitinib combined with vinblastine and azacitidine^22^. In a further study that included patients with genetic or immunohistochemical evidence of JAK/STAT activation, the efficacy of ruxolitinib was similar to that of agents approved for the treatment of relapsed/refractory T-cell lymphoma^23^, suggesting that ruxolitinib has therapeutic potential in A-TLL with JAK/STAT activation.

Artesunate inhibited the RNA expression of *IL-1β* and *TNF-α* and protein expression of P-JAK2 and P-STAT5 in Jurkat cells but had no significant effect on RNA expression of *JAK2* and *STAT3* or protein expression of JAK2 and STAT5. The effect of artesunate on the JAK/STAT pathway might be mediated through its influence on the activation state of the JAK/STAT pathway at the protein level but not its effects on RNA transcription. This is consistent with the results of Su et al.^12^ with regard to the effects of artesunate on JAK2 and P-JAK2 expression. The effect of artesunate on expression of STAT5 protein aligns with the finding by Kim et al. that this drug only inhibits the activation state of STAT5^24^. In a study by Jochims et al., the cytotoxic effects of artesunate were mainly dependent on the intracellular iron concentration, induction of oxidative stress, and upregulation of HO-1, which coincide with the characteristics of tumor cells, resulting in highly selective destruction of tumor and inflammatory cells and less toxicity to normal cells^25,26^. The high selectivity of artesunate for tumor cells makes it a potentially effective and safe therapeutic agent for T-ALL.

In this study, treatment of Jurkat cells with a combination of artesunate and ruxolitinib resulted in similar effects as treatment with the individual drugs, both of which inhibited the growth of Jurkat cells by suppressing the phosphorylation of JAK2 and STAT5. The growth-inhibiting and apoptosis-promoting effects of the two drugs in combination were stronger than those of either drug alone. However, the drugs had an antagonistic effect at low concentrations but a synergistic effect at high concentrations. Meanwhile, p-STAT5 expression was significantly less in the combination group than in the monotherapy groups, with no significant between-group differences observed in the expression of other proteins. After combined treatment with artesunate and ruxolitinib, p-STAT5 expression in Jurkat cells was significantly decreased, whereas expression of p-JAK2 remained unchanged; furthermore, the JAK2 phosphorylation level was reduced after treatment with artesunate without alterations in its transcriptional level. Artesunate and ruxolitinib might exert synergistic effects through pathway-specific regulation at a downstream target, via which artesunate can increase phosphatase activity to accelerate dephosphorylation of p-STAT5, without having an effect on p-JAK2 expression upstream. Notably, the unchanged transcriptional levels clearly indicate that artesunate acts at the non-transcriptional stage, namely, by focusing on post-translational modification. This non-transcriptional regulatory mode has high specificity. Specifically, artesunate does not interfere with initiation of transcription, synthesis of mRNA, or stability of target genes, but directly targets the dynamic equilibrium of protein phosphorylation/dephosphorylation, helping to avoid extensive perturbation of the global cellular transcriptome and reduce off-target toxicity, thereby ensuring the precision and safety of combined therapy. Furthermore, the phenomenon whereby JAK2 transcription was upregulated while its protein expression was downregulated after ruxolitinib monotherapy and artesunate–ruxolitinib combination treatment reflects the dissociation effect between gene transcription and protein translation/degradation and reveals the multilevel specificity of drug regulation in the JAK2/STAT5 pathway. The increased *JAK2* RNA level is a compensatory transcriptional response of cells to the inhibited protein function that aims to compensate for the insufficient pathway activity by increasing mRNA synthesis; in contrast, the decreased protein expression is the result of direct drug action at the post-translational stage, and the protein degradation or translation inhibition effect mediated by drugs is significantly stronger than the promoting effect of transcriptional compensation. This dissociation pattern further confirms that regulation of the pathway by artesunate and ruxolitinib does not depend on initiation of transcription, but targets post-translational modification and protein metabolism processes. While avoiding perturbation of the global transcriptome, it achieves precise blocking of the pathway, providing key experimental evidence for the safety and specificity of combination therapy.

This study had some limitations. First, the number of T-ALL samples included in the immunohistochemical experiments was insufficient to accurately ascertain the true expression of JAK2/STAT5 and its phosphorylation in T-ALL. Therefore, our findings cannot support extensive conclusions regarding the clinical treatment of T-ALL with artesunate and ruxolitinib. Second, the drug experiments were conducted only in a Jurkat cell line, so their results cannot be extrapolated to human patients. These limitations need to be considered carefully when extrapolating our results to clinical practice. Future studies should prioritize the use of multiple cell lines to clarify the effects of artesunate and ruxolitinib on different cell subtypes and confirm their efficacy. Furthermore, in vivo experiments should be conducted in patient-derived xenograft models, and well-designed prospective clinical trials are needed to evaluate the safety and effectiveness of artesunate and ruxolitinib in clinical practice.

## Conclusion

In this study, we investigated the growth-inhibiting and apoptosis-promoting effects of ruxolitinib and artesunate alone and in combination on Jurkat cells in vitro. Combination of (1) 7.5 µmol artesunate and 40 µmol ruxolitinib, (2) 15 µmol artesunate and 10, 20, or 40 µmol ruxolitinib, and (3) 20 µM artesunate and 30 µM ruxolitinib have synergistic effects, promoting apoptosis and inhibiting cell proliferation more effectively than either agent alone. The combined effects of these drugs might reflect inhibition of the phosphorylation of proteins involved in the JAK/STAT pathway. Our findings have potential implications for T-ALL with JAK/STAT mutations. Mechanistic investigations in this study were confined to Jurkat cells, so validation is required in additional cell lines. Animal studies and clinical trials will be needed to obtain more robust evidence.

This study was supported by the Traditional Chinese Medicine Scientific Research Project (2023004).

**Table 1:** Primer sequences used for reverse transcription-quantitative polymerase chain reaction.

Primer	Sequence
ACTIN	5′- CTCTTCCAGCCTTCCTTCCT-3′
	5′- AGCACTGTGTGTTGGCGTACAG-3′
*JAK2*	5′- CCAGATGGAAACTGTTCGCTCAG-3′
	5′- GAGGTTGGGTACATCAGAAACACC-3′
*STAT3*	5′- CTTTGAGACCGAGGTGTATCACC-3′
	5′- GGTCAGCATGTTGTACCACAGG-3′
*TNF-α*	5′- CAGATGTGGGGGTGTGAGAAGAG-3′
	5′- TCCTCCACCCTTCCCTTGAG-3′
*IL-β*	5′- TAGCTTCCCCATGACGGCTA-3′
	5′- CAGGCTGCTCTGGGGATTCTC-3′

**Table 2:** IC_50_ values for Artesunate and ruxolitinib in Jurkat cells.

Drug	24 h IC50 Value	48 h IC50 Value
ART	12.86µM	5.412µM
RUX	30.55µM	15.51µM

ART, artesunate; RUX, ruxolitinib; IC_50_, half-maximal inhibitory concentration.

**Table 3:** Combination index data for Non-Constant combo (artesunate + ruxolitinib).

Dose ART(µM)	Dose RUX(µM)	Effect	CI
7.5	10	0.6171	1.18654
7.5	20	0.5518	1.07903
7.5	40	0.4179	0.79481
15	10	0.5138	0,96857
15	20	0.4681	0.91652
15	40	0.3999	0.87563
20	30	0.39702	0.82647

ART, artesunate; CI, combination index; RUX, ruxolitinib.

## Supplementary Information

Below is the link to the electronic supplementary material.

Supplementary Material 1

Supplementary Material 2

Supplementary Material 3

Supplementary Material 4

Supplementary Material 5

## Data Availability

The datasets used and/or analyzed in the current study are available from the corresponding author upon reasonable request.
