# Supplementary figures and images for: Combination of artesunate and ruxolitinib suppresses T cell leukemia/lymphoma proliferation via the JAK STAT pathway
Source: Sci Rep. 2026 Feb 11;16:8354. doi: 10.1038/s41598-026-39393-8 (PMC12966290; doi:10.1038/s41598-026-39393-8)

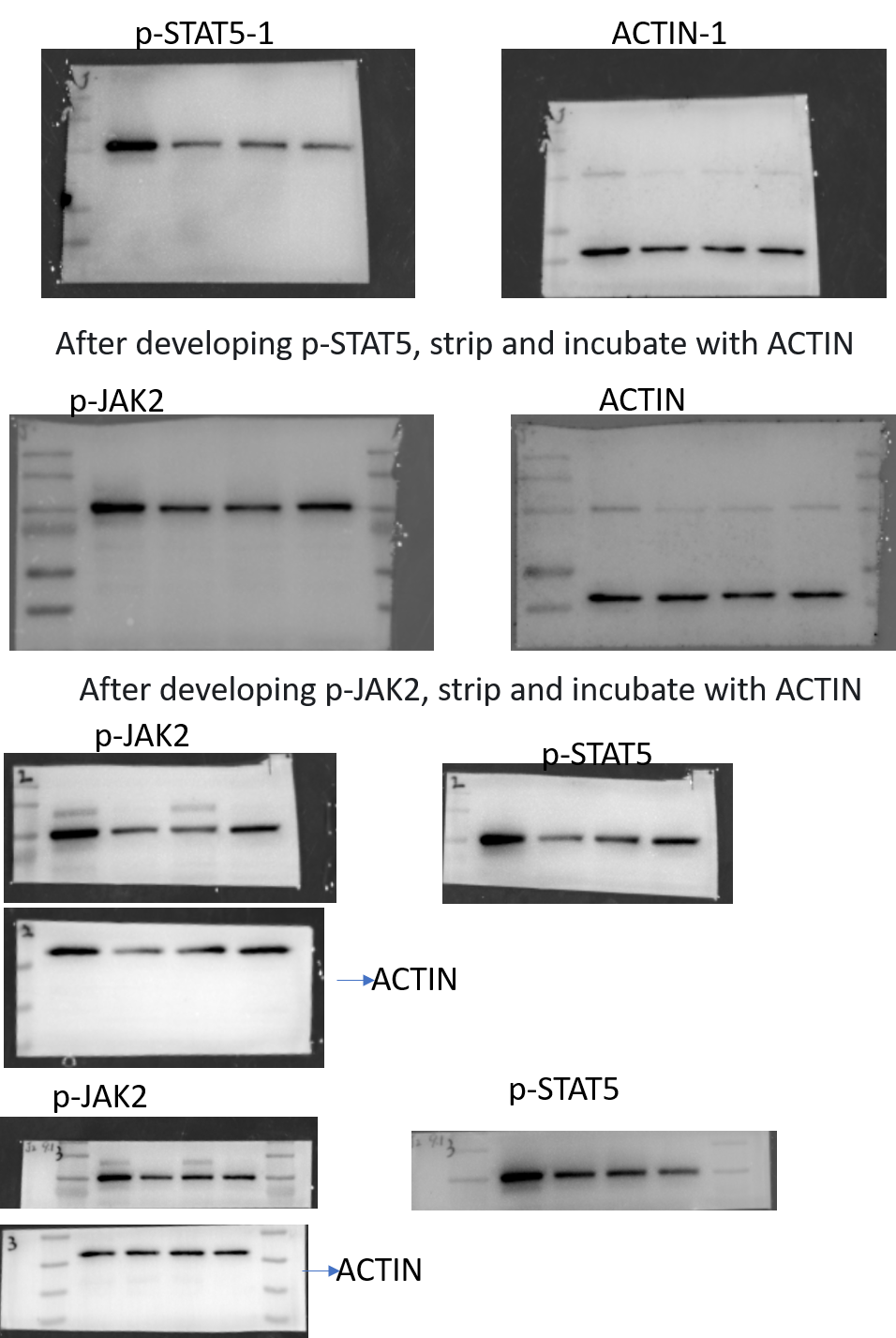

Supplement: Supplementary file 2 — Supplementary Material 2 [file 41598_2026_39393_MOESM2_ESM.png]
